# Afriplex GRT^TM^ extract attenuates hepatic steatosis in an *in vitro* model of NAFLD

**DOI:** 10.1371/journal.pone.0297572

**Published:** 2024-04-17

**Authors:** Kwazi Gabuza, Thendo I. Mabuda, Oelfah Patel, Noxolo Khuboni, Ruzayda van Aarde, Sylvia Riedel, Nonhlakanipho F. Sangweni, Shantal Windvogel, Rabia Johnson, Christo J. F. Muller

**Affiliations:** 1 Biomedical Research and Innovation Platform, South African Medical Research Council, Parow Valley, Cape Town, South Africa; 2 Department of Biotechnology, University of the Western Cape, Bellville, Cape Town, South Africa; 3 Department of Biochemistry and Microbiology, University of Zululand, eMpangeni, South Africa; 4 Centre for Cardio-Metabolic Research in Africa (CARMA), Division of Medical Physiology, Faculty of Medicine and Health Sciences, Stellenbosch University, Tygerberg, Cape Town, South Africa; University of Brescia: Universita degli Studi di Brescia, ITALY

## Abstract

**Background:**

Currently, it is acknowledged that vitamin E, insulin sensitizers and anti-diabetic drugs are used to manage non-alcoholic fatty liver disease (NAFLD), however, these therapeutic interventions harbour adverse side effects. Pioglitazone, an anti-diabetic drug, is currently the most effective therapy to manage NAFLD. The use of natural medicines is widely embraced due to the lack of evidence of their negative side effects. Rooibos has been previously shown to decrease inflammation and oxidative stress in experimental models of diabetes, however, this is yet to be explored in a setting of NAFLD. This study was aimed at investigating the effects of an aspalathin-rich green rooibos extract (Afriplex GRT^TM^) against markers of hepatic oxidative stress, inflammation and apoptosis in an *in vitro* model of NAFLD.

**Methods:**

Oleic acid [1 mM] was used to induce hepatic steatosis in C3A liver cells. Thereafter, the therapeutic effect of Afriplex GRT^TM^, with or without pioglitazone, was determined by assessing its impact on cell viability, changes in mitochondrial membrane potential, intracellular lipid accumulation and the expression of genes and proteins (*ChREBP*, *SREBF1*, *FASN*, *IRS1*, *SOD2*, Caspase-3, GSTZ1, IRS1 and TNF-α) that are associated with the development of NAFLD.

**Results:**

Key findings showed that Afriplex GRT^TM^ added to the medium alone or combined with pioglitazone, could effectively block hepatic lipid accumulation without inducing cytotoxicity in C3A liver cells exposed oleic acid. This positive outcome was consistent with effective regulation of genes involved in insulin signaling, as well as carbohydrate and lipid metabolism (*IRS1*, *SREBF1* and *ChREBP*). Interestingly, in addition to reducing protein levels of an inflammatory marker (TNF-α), the Afriplex GRT^TM^ could ameliorate oleic acid-induced hepatic steatotic damage by decreasing the protein expression of oxidative stress and apoptosis related markers such as GSTZ1 and caspase-3.

**Conclusion:**

Afriplex GRT^TM^ reduced hepatic steatosis in oleic acid induced C3A liver cells by modulating *SREBF1*, *ChREBP* and *IRS-1* gene expression. The extract may also play a role in alleviating inflammation by reducing TNF-α expression, suggesting that additional experiments are required for its development as a suitable therapeutic option against NAFLD. Importantly, further research is needed to explore its antioxidant role in this model.

## Introduction

To date, approximately 1.8 billion of the global population account for non-alcoholic fatty liver disease (NAFLD) cases, most of which are largely contributed to the increased prevalence of obesity and diabetes [1]. Notably, NAFLD is a result of the abnormal increase in free fatty acid (FFA) diffusion within hepatic cells [2]. FFA molecules are important for numerous cellular activities such as, cellular membrane synthesis, intracellular signaling and energy storage in adipose tissues. As such, it is currently acknowledged that an uncontrolled build-up of FFAs may lead to insulin resistance, through the disruption of metabolic signaling. The liver plays a key role in lipid metabolism through the transportation, storage and intake of FFAs during an abundance or shortage of FFA molecules within the human body. The compression of hepatocytes during the progression of NAFLD leads to an abnormal inflammatory response due to the increased infiltration of FFAs, resulting in hepatitis [3, 4]. Beyond a state of persistent inflammation [3, 4], hepatic steatosis is defined as the histological change where the liver fat content exceeds the total liver mass by 5% [5]. Numerous studies have demonstrated that the reduction in mitochondrial β-oxidation and *de novo* lipogenesis, which may be facilitated by enhanced FFA delivery and poor integration of lipids by the liver may trigger hepatic steatosis [6–8]. In addition, the constant infiltration of FFAs into the liver has been reported to induce lipoperoxidative stress and hepatic damage [8]. Moreover, β-oxidation and esterification, which are needed for hepatic ATP and triglyceride regulation, are said to be key mediators in the formation of very-low-density lipoprotein (VLDL) particles that are implicated in the development of insulin resistance [9].

As recently reviewed [10], the presence of insulin resistance in combination with inflammation, has been shown to accelerate the rate of hepatic apoptosis via the activation of pro-apoptotic and pro-oxidative pathways. This then leads to structural modifications which may impair the proper functioning of organs surrounding the hepatic system. Furthermore, the deterioration of the hepatic system, due to excessive lipid accumulation and poor insulin sensitivity, causes severe hepatitis [11]. Worldwide, NAFLD has been projected to become the most common chronic liver disorder leading to hepatocellular carcinoma [1]. Several therapeutic strategies, such as thiazolidinediones (TZDs), have been used to alleviate the disease burden caused by NAFLD [12, 13]. Briefly, TZDs, such as pioglitazone and rosiglitazone, are classified as anti-diabetic agents, with potent anti-inflammatory and insulin sensitizing effects [13]. Mechanistically, these drugs have been shown to effectively reduce hepatic fat content by 30–50% in patients with steatohepatitis [5]. In a placebo-controlled trial study, administration of TZDs was linked with improved insulin sensitivity, that was consistent with histologic improvements in subjects with nonalcoholic steatohepatitis [14]. While these drugs are very potent, several countries have withdrawn their use as therapeutics as they have been shown to infer contraindications, such as the development of bladder tumors, jaundice and blurred vision, among other side effects [15].

Currently, numerous plant based therapies have be investigated for their therapeutic properties against the development of NAFLD [16–18]. Of interest to this study is *Aspalathus linearis*, which is a flowering bush endemic to South Africa, is commonly known as rooibos. While prominently consumed as a herbal tea rich in antioxidants, rooibos has been recently developed into a potent nutraceutical specifically targeted at alleviating and reducing the risk of developing diabetes related co-morbidities [19–23]. The pharmaceutical benefits of rooibos are largely attributed to its polyphenolic content which includes the anti-diabetic aspalathin (a *C*-linked dihydrochalcone glucoside) and anti-inflammatory Z-2-(β-D-glucopyranosyloxy)-3-phenylpropenoic acid (PPAG) [24]. Recent findings of a green rooibos extract’s effect on the hepatic morphometric quantification of steatosis, suggest that it could be useful in attenuating NAFLD [25]. As such, we investigated the mechanism by which a green rooibos extract (Afriplex GRT^TM^) alleviates oleic acid-induced hepatic steatosis in an *in vitro* C3A model.

## Materials and methods

### Chemical composition of Afriplex GRT^TM^ extract

Afriplex GRT^TM^ is an active pharmaceutical ingredient that was developed by Afriplex Pharmaceuticals PTY (LTD) (Paarl, South Africa) in partnership with the Biomedical Research and Innovation Platform (BRIP) of the South African Medical Research Council (SAMRC, Cape Town, South Africa). The extract used in this study was previously characterized for its phenolic content shown to contain 12.8% aspalathin [22]. The extract can be purchased from Afriplex Pharmaceuticals PTY (LTD), 10 De Vreugde Crescent, Paarl, South Africa, Tel: +2721 872 4930. The details about the extract’s physical and chemical properties are contained in the supporting information **S5 File** Afriplex GRT^TM^ product safety data sheet and the link to the full product safety data sheet has been provided. The lists of units of measurements and abbreviations used are available in Lists 1–2, with the information about the used materials and kits in Table 1 in S3 File Measurement units, abbreviations, chemicals and kits.

### Experimental design

C3A liver cells, an immortalized human liver cell line sub-clone derived from HepG2 (HepG2/C3A) hepatocytes, were utilized in this study as a representative liver cell line to experimentally induce and treat hepatic steatosis. The HepG2/C3A cells have a closer phenotype to human hepatocytes, can grow on glucose deficient media and their use in liver spheroids demonstrates high sensitivity to drug induced liver injury [26]. Cells were obtained from the American Type Culture Collection [ATCC CRL-10741] (Manassas, VA, USA). Cells were cultured in pre-warmed (37°C) Eagle’s essential minimal medium (EMEM) containing 1 mM sodium pyruvate and non-essential amino acids (NEAA) (Lonza, Basel, Switzerland), supplemented with 10% fetal bovine serum (FBS) (Highveld Biological, Lyndhurst, South Africa), 1% of L-glutamine and 19.5 mM glucose, incubated at 37°C in humidified air with 5% CO_2_. The cultured cells were refreshed every second day until they reached the targeted confluency (80%), and cell suspensions were seeded at a standardized seeding density of 1.0 x 10^5^ cells per mL.

To establish a model of steatosis, the cultured cells were exposed to oleic acid, purchased from Sigma-Aldrich Co. (St. Louis, MO, USA), at an optimized concentration [1 mM]. The induction media was prepared by adding the oleic acid in cell culture media supplemented with 2% bovine serum albumin (BSA), the solution was conjugated at 37°C for 2 hrs, and the cells incubated at 37°C in humidified air with 5% CO_2_ for 24 hrs. Following incubation, the experimental plates were mono-treated with pioglitazone [30 μM], purchased from Sigma-Aldrich Co. (St. Louis, MO, USA), and Afriplex GRT^TM^ [1, 10 and 100 μg/mL]. Co-treatments were also assessed by combining Afriplex GRT^TM^ [1, 10 and 100 μg/mL] with pioglitazone [30 μM] and the treatments were incubated at 37°C in humidified air with 5% CO_2_ for 24 hrs. The determination of the non-toxic amount of Afriplex GRT^TM^ used were based on the previous study that was conducted in our lab [27]. Biochemical and cytotoxicity tests were conducted to confirm the impact of treatment compounds on *in vitro* markers of hepatic steatosis. Following treatment, cells were also harvested for mRNA expression (qRT-PCR) and protein expression (Western blot) analysis.

### Lipid content analysis (Oil red O assay)

The lipid content following exposure of C3A cells to oleic acid and the potential lipid-lowering effect of treatment were determined using an Oil red O (ORO) based staining assay according to manufacturer’s instructions (Sigma-Aldrich Co., St. Louis, MO, USA). Following treatment, the C3A liver cells were briefly fixed using 10% formalin in 96-well tissue culture plates for fifteen minutes at room temperature. Once fixed the cells were washed with 100 μL phosphate-buffered saline (PBS) (Lonza, Basel, Switzerland). A 0.7% ORO working solution was then added to the cells and subsequently incubated for thirty minutes at room temperature. The ORO stain was aspirated, and the stained cells were rinsed several times with distilled water and visualized under an inverted light microscope with a digital camera (Olympus CK x31, Tokyo, Japan). The cells were captured during visualization and then the distilled water was aspirated, and absolute isopropanol was added to the cells to extract the stained lipids. The extracted stain was transferred into a fresh 96-well assay plate and read at an absorbance of 490 nm, using a plate reader ELx800 (BioTek^®^, Winooski, VT, USA), which is equipped with Gen 5 software for data acquisition (BioTek Instruments Inc., Winooski, VT, USA).

After ORO extraction, the cells were washed with 70% ethanol and were subsequently stained with 0.5% crystal violet (CV) working solution (Sigma-Aldrich Co., St. Louis, MO, USA). The cells were then incubated for five minutes at room temperature. The CV stain was then washed with PBS and 70% ethanol was added to extract the CV stain from the cells. The extracted stain was therefore transferred into a fresh 96-well assay plate and read on a ELx800 plate reader at an absorbance of 570 nm.

### Mitochondrial dehydrogenase activity (MTT assay)

The mitochondrial succinate dehydrogenase activity was analyzed to quantify cell viability using a 3-[4,5-dimethylthiazol-2-yl]-2,5-diphenyltetrazolium (MTT) dye (Sigma-Aldrich Co., St. Louis, MO, USA), as described by Mosmann in 1983 [28]. The MTT assay was conducted in triplicate on 96-well tissue culture plates by incubating the treated cells with an MTT reagent (2 mg/ml) at 37°C for thirty minutes. After incubation, 200 μL dimethyl sulfoxide (DMSO) (Sigma-Aldrich Co., St. Louis, MO, USA), and 25 μL Sorenson’s buffer was added into each well, and the plate was kept at room temperature, wrapped in aluminum foil to block out light. The plate was subsequently read using a ELx800 plate reader at an absorbance reading of 570 nm.

### Mitochondrial membrane potential (JC-1 assay)

Changes in mitochondrial membrane potential of the cells following treatment were analyzed to determine mitochondrial depolarization using a lipophilic cationic fluorescent dye; 5, 5’, 6, 6’-Tetrachloro-1, 1’, 3, 3’-tetraethylbenzimidazolylcarbocyanine iodide (JC-1) purchased from Sigma-Aldrich Co. (St. Louis, MO, USA). The JC-1 dye fluoresces red (λex 596 nm) and green (λex 520 nm) to indicate healthy or depolarized cells respectively, described by Sivandzade *et al*. (2019) [29]. The JC-1 assay was conducted in triplicate on 24-well tissue culture plates by incubating the treated cells with 2 μM of the JC-1 stain at 37°C for thirty minutes. Following incubation, the cells were washed with 500 μL PBS and the stained cells were visualized and imaged using a Nikon Eclipse Ti/S inverted fluorescence microscope with digital camera (Nikon, Minato City, Tokyo, Japan). The images taken using microscopy were evaluated to find the proportions of red to green fluorescence using the ImageJ32.app software application (imagej.nih.gov/ij/list.html) through densitometric analysis.

### mRNA extraction and quantification (qRT-PCR)

Total cellular mRNA (messenger ribonucleic acid) was extracted from the treated cells harvested from 6-well tissue culture plates, using a RNeasy mini kit (ThermoFischer Scientific™, Waltham, Massachusetts, USA) as per the manufacturer’s instructions. Purified mRNA was thereafter reverse transcribed into single‐stranded complementary DNA (cDNA) using the High-Capacity cDNA reverse transcription kit (Applied Biosystems, Foster City, CA, USA), according to the manufacturer’s instructions. Quantitative real time polymerase chain reaction (qRT-PCR) was performed using TaqMan probes (Applied Biosystems, Foster City, CA, USA) to detect the relative expression of genes involved in insulin signaling (Insulin receptor substrate 1 (*IRS1*)), lipid and glucose metabolism (Sterol regulatory element-binding transcription factor 1 (*SREBF1*), fatty acid synthase (*FASN*) and carbohydrate response element binding protein (*ChREBP*)) and oxidative stress (Superoxide dismutase 2 (*SOD2*), respectively. The probes and the conditions used for gene expression studies are detailed in Tables 1 and 2 in S2 File qRT-PCR TaqMan probes and conditions. Reactions were run using an ABI 700 Sequence Detection System (Applied Biosystems, Foster City, CA, USA), where *β-Actin* was used for house-keeping and the relative change in expression was determined using a standard curve.

### Protein extraction and Western blot analysis

Total protein was extracted from the treated cells, harvested from 6-well tissue culture plates, using an Invitrogen™ Tissue Extraction Reagent I (ThermoFischer Scientific™, Waltham, Massachusetts, USA) as described by Johnson *et al*. in 2016 [30]. Protein concentrations were determined using a Pierce™ BCA Protein Assay Kit (ThermoFischer Scientific™, Waltham, Massachusetts, USA) as per the manufacturer’s instructions. Equal amounts of protein were subjected to sodium dodecyl sulfate-polyacrylamide gel electrophoresis (SDS PAGE) and subsequently electro-transferred onto a polyvinylidene fluoride (PVDF) membrane. The membranes were blocked for two hours in 5% fat-free milk at room temperature and subsequently incubated overnight at 4°C with primary antibodies (1:1000) (Cell Signaling Technology, Danvers, MA, USA), (Santa Cruz Biotechnology, Dallas, Texas, USA) & (Abcam, Cambridge, UK) specific to proteins involved in: insulin signaling (IRS1), oxidative stress (glutathione S-transferase zeta 1 (GSTZ1), inflammatory response (tumor necrosis factor-alpha (TNF-α) & (nuclear factor kappa B (NFκB), apoptotic pathways (caspase-3 (CASP3), fatty acid synthesis pathways (fatty acid synthase (FASN) and beta actin (β-actin) for house-keeping. The details of antibodies that were used are given in Table 1 in S4 File List of antibodies. Following incubation, the membranes were washed three times at 10 min intervals with 1 x tris buffered saline–tween20® (TBST), after which the membranes were incubated for ninety minutes with anti-rabbit or anti-mouse secondary antibodies (1:4000) (Cell Signaling Technology, Danvers, MA, USA). The immunoblots were visualized using a LumiGLO Chemiluminescent Substrate Kit (ThermoFischer Scientific™, Waltham, Massachusetts, USA) as per manufacturer’s instructions and bands were visualized and captured on a ChemiDoc MP (Bio-Rad, Hercules, USA). The intensity of the bands were quantified using the ImageLab™ software version 6.0.1 for Macintosh, Hercules, USA, www.bio-rad.com.

### Statistical analysis

The data is presented as mean ± standard error of the mean (SEM). The significance of the raw data from the biochemical assays were analyzed using One-way ANOVA followed by Tukey’s multiple comparisons test, using the GraphPad Prism^®^ software, version 7.00 for Macintosh, La Jolla California USA, www.graphpad.com. Differences were statistically significant when p-value < 0.05.

## Results

### Lipid intake analysis

**Fig 1:**
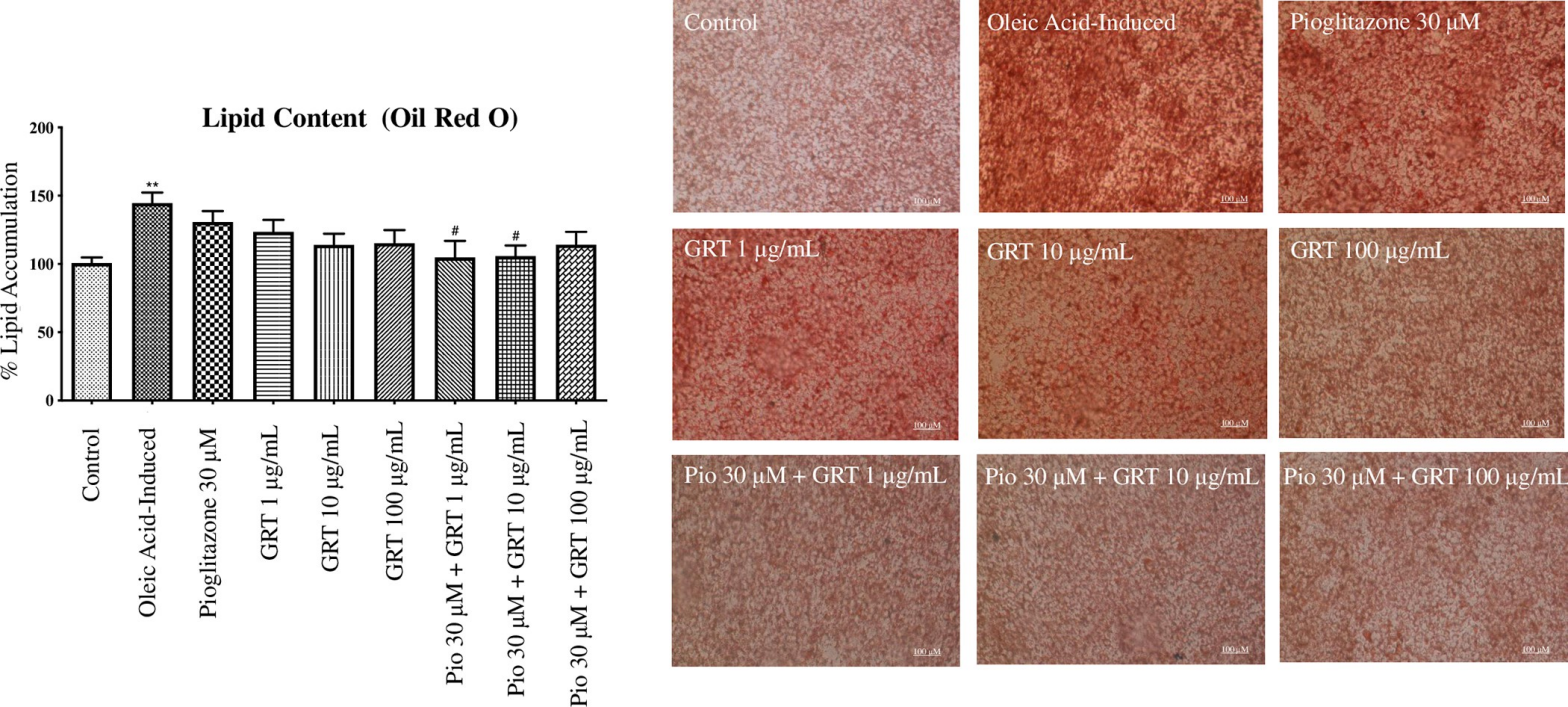
Oil Red O assay, showing the effects of steatosis induction [1 mM oleic acid] and treatment effects of pioglitazone and Afriplex GRT^TM^ (GRT), on intracellular lipid content. Images are a representation of one experiment, captured at 200x magnification using a Nikon Eclipse Ti/S inverted light microscope with a digital camera (scale represents: 100 μm). Histogram illustrates spectrophotometric data of three independent repeats of the ORO assay. Data represents mean ± SEM (n = 3), where **P < 0.01 when compared to the control and ^#^P < 0.05, when compared to the oleic acid-induced cells respectively.

A significant accumulation in lipid content with the oleic acid-induced cells (p < 0.01) was observed (**Fig 1)** compared to the untreated cells (control). Pioglitazone and Afriplex GRT^TM^ showed no significant changes in lipid content when administered as mono treatments. Interestingly, when compared to the oleic acid-induced cells, pioglitazone (30 μM) in combination with Afriplex GRT^TM^ at both 1 μg/mL and 10 μg/mL significantly decreased lipid content (p < 0.05) (**Fig 1**).

### Mitochondrial activity and membrane potential

**Fig 2:**
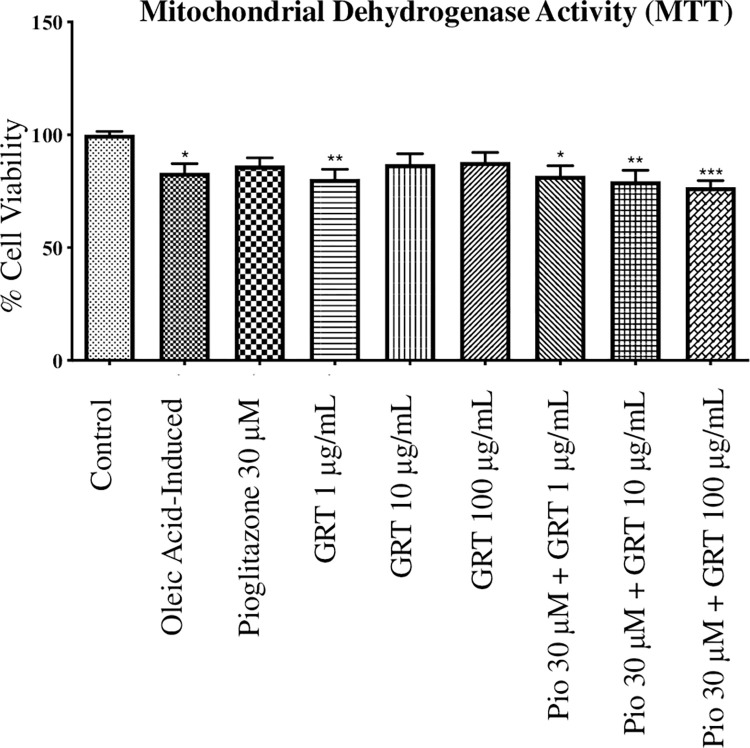
MTT assay, showing the effects of steatosis induction [1 mM oleic acid] and treatment effects of pioglitazone and Afriplex GRT^TM^ (GRT), on mitochondrial activity. Data represents mean ± SEM (*n* = 3), where *P < 0.05, **P < 0.01 and ***P < 0.001 when compared to the control.

A significant reduction in mitochondrial activity, to indicate reduced cell viability, was observed for the oleic acid-induced cells (p < 0.05) when compared to the control (**Fig 2**). Afriplex GRT^TM^ (1 μg/mL) as a mono- and co-treatment with pioglitazone (30 μM) reduced mitochondrial activity (**Fig 2**, p < 0.01). Similarly, the combination of pioglitazone (30 μM) with Afriplex GRT^TM^ at 10 μg/mL (p < 0.01) and 100 μg/mL (p < 0.001) also showed significant reductions in mitochondrial activity when compared to the control. Despite the significant decrease in mitochondrial activity, the treated cells retained mitochondrial activity at 70%, suggesting that both the induction period with oleic acid and subsequent treatment with Afriplex GRT^TM^ and/or pioglitazone was not cytotoxic.

**Fig 3:**
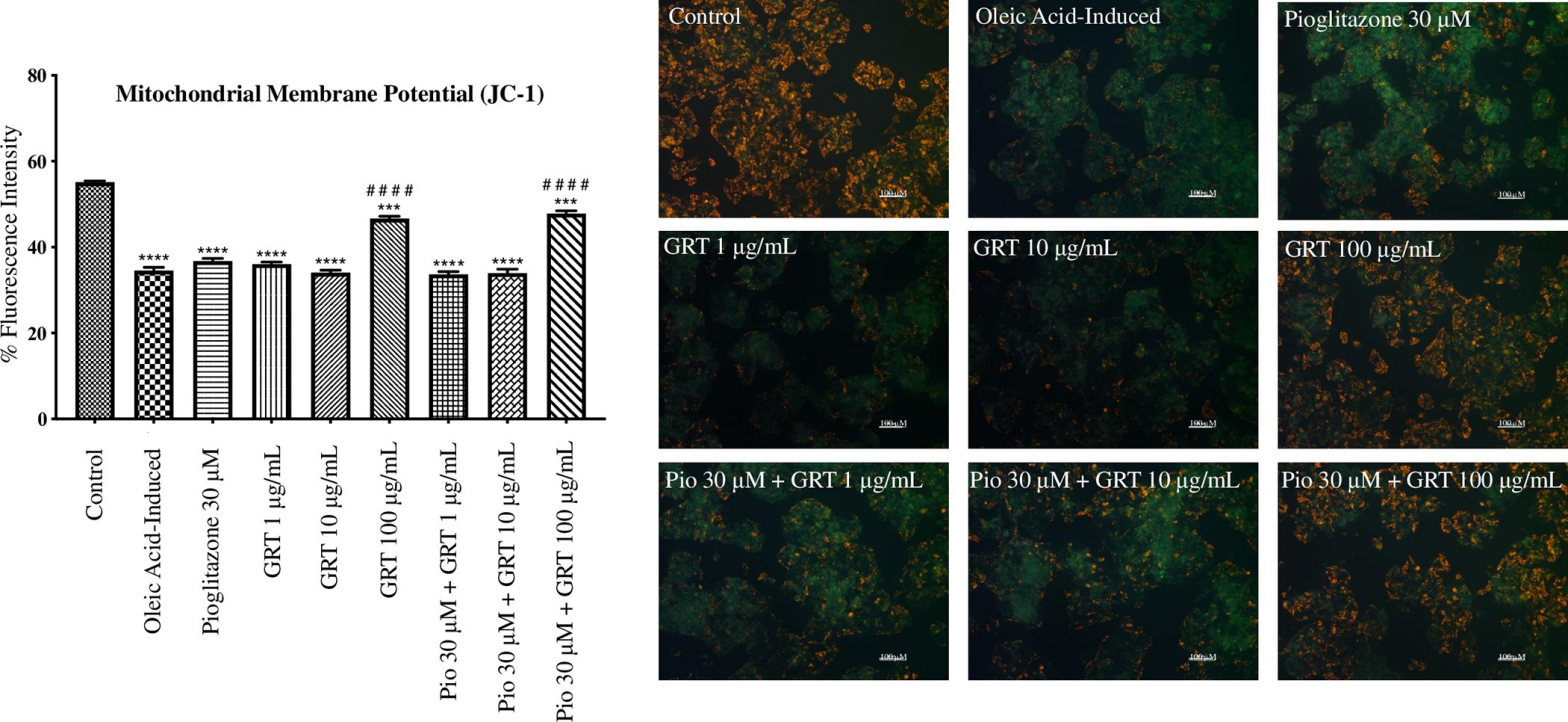
JC-1 assay, showing the effects of steatosis induction [1 mM oleic acid] and treatment effects of pioglitazone and Afriplex GRT^TM^ (GRT), on mitochondrial membrane potential. Images are a representation of one experiment, captured at a 200x magnification using a Nikon Eclipse Ti/S inverted fluorescence microscope with a digital camera (scale represents: 100 μm). Histogram illustrates densitometric quantification of the JC-1 assay. The red fluorescence shown in the images was quantified using ImageJ32.app software. Data represents mean ± SEM (*n* = 3), where ***P < 0.001 and ****P < 0.0001 when compared to the control, and ^####^P < 0.0001, when compared to the oleic acid-induced cells, respectively.

Densitometric data illustrated by the histogram in **Fig 3** showed a significant reduction in red fluorescence, as an indication of mitochondrial depolarization, when comparing the oleic acid-induced cells to the control (p < 0.0001). This significant reduction in red fluorescence was also seen across the various treatments. Treatment with Afriplex GRT^TM^ (100 μg/mL) as a mono-treatment and treatment with pioglitazone 30 μM in combination with Afriplex GRT^TM^ 100 μg/mL significantly improved the condition when compared to the control (p < 0.001). Interestingly, treatment with Afriplex GRT™ (100 μg/mL) as a mono-treatment and in combination with pioglitazone (30 μM) showed a significant increase in red fluorescence when compared to the oleic acid-induced cells (p < 0.0001), suggesting that treatment with this extract at a concentration of 100 μg/mL restored the mitochondrial membrane potential relative to the oleic acid-induced cells with no additive effect by its combined administration with pioglitazone (30 μM).

### mRNA expression

**Fig 4:**
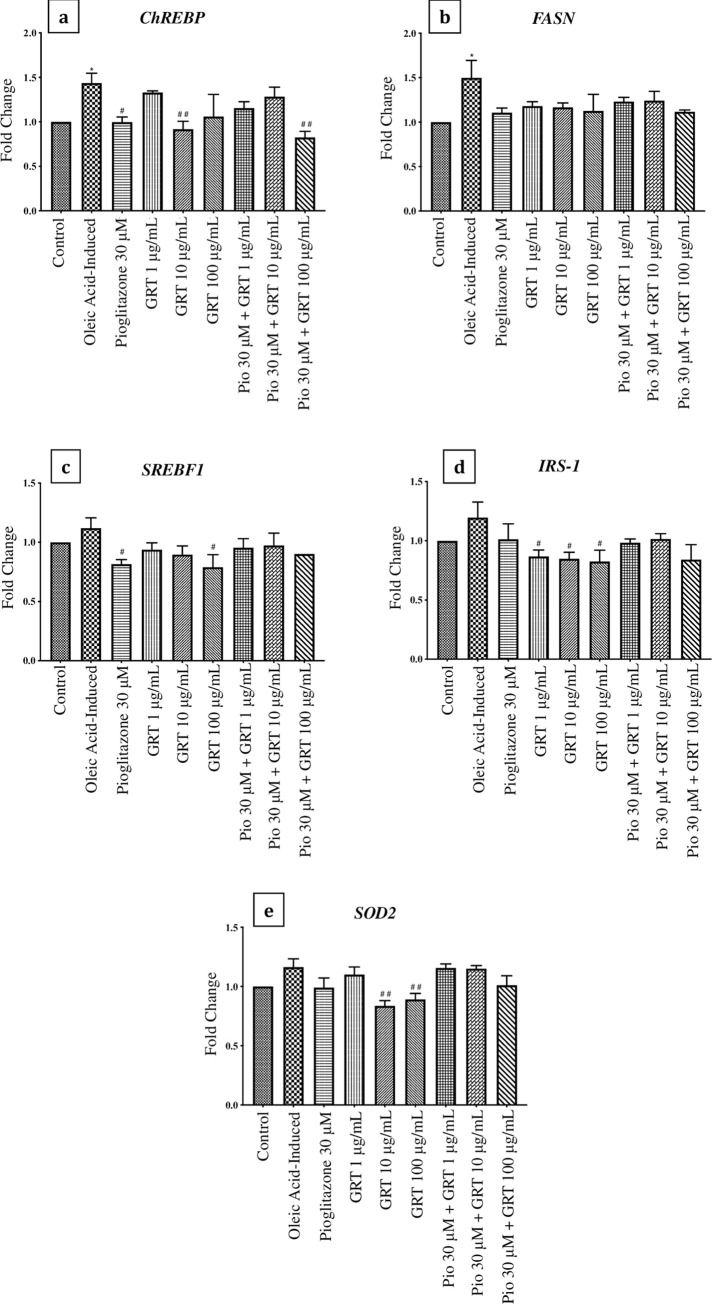
mRNA expression of C3A cell-lysates normalized to *β-actin*, showing the effects of steatosis induction [1 mM oleic acid] and treatment effects of pioglitazone and Afriplex GRT^TM^. Data represents mean ± SEM (*n* = 3), where *P < 0.05, when compared to the control, and ^#^P < 0.05, ^##^P < 0.01 when compared to the oleic acid-induced cells. Histograms represent the mRNA expressions of; (**a**) *ChREBP* (**b**) *FASN*, (**c**) *SREBF1*, (**d**) *IRS-1* & (**e**) *SOD2* respectively. **ChREBP**—carbohydrate response element binding protein, **FASN—**fatty acid synthase, **SREBF1—**sterol regulatory element-binding transcription factor 1, **IRS-1—**insulin receptor substrate 1, **SOD2—**superoxide dismutase 2.

The mRNA expression of genes involved in lipid metabolism (*ChREBP*, *FASN* and *SREBF1*) (**Fig 4**) was up-regulated by oleic acid when compared to the control, with significance observed in the expression of *ChREBP* (p < 0.05) and *FASN* (p < 0.05). Pioglitazone (30 μM) (p < 0.05) and Afriplex GRT^TM^ (10 μg/mL) (p < 0.01) as mono-treatments, significantly down-regulated *ChREBP* gene expression when compared to the oleic acid-induced cells. The combined treatment of pioglitazone (30 μM) and Afriplex GRT^TM^ (100 μg/mL) showed a significant down-regulation in the expression of *ChREBP* (p < 0.01), when compared to the oleic acid-induced cells (**Fig 4A**). The mRNA expression of *SREBF1* showed a significant down-regulation by treatment with pioglitazone (30 μM) and Afriplex GRT^TM^ (100 μg/mL) as mono-treatments when compared to the oleic acid-induced cells (p < 0.05) (**Fig 4C**). Treatment with Afriplex GRT^TM^ significantly down-regulated the mRNA expression of *IRS-1*, when compared to the oleic acid-induced cells (p < 0.05) (**Fig 4D**). *SOD2* mRNA expression was also significantly down-regulated by treatment with Afriplex GRT^TM^ (10 μg/mL and 100 μg/mL) when compared to the oleic acid-induced cells (p < 0.01) (**Fig 4E**).

### Protein expression

**Fig 5:**
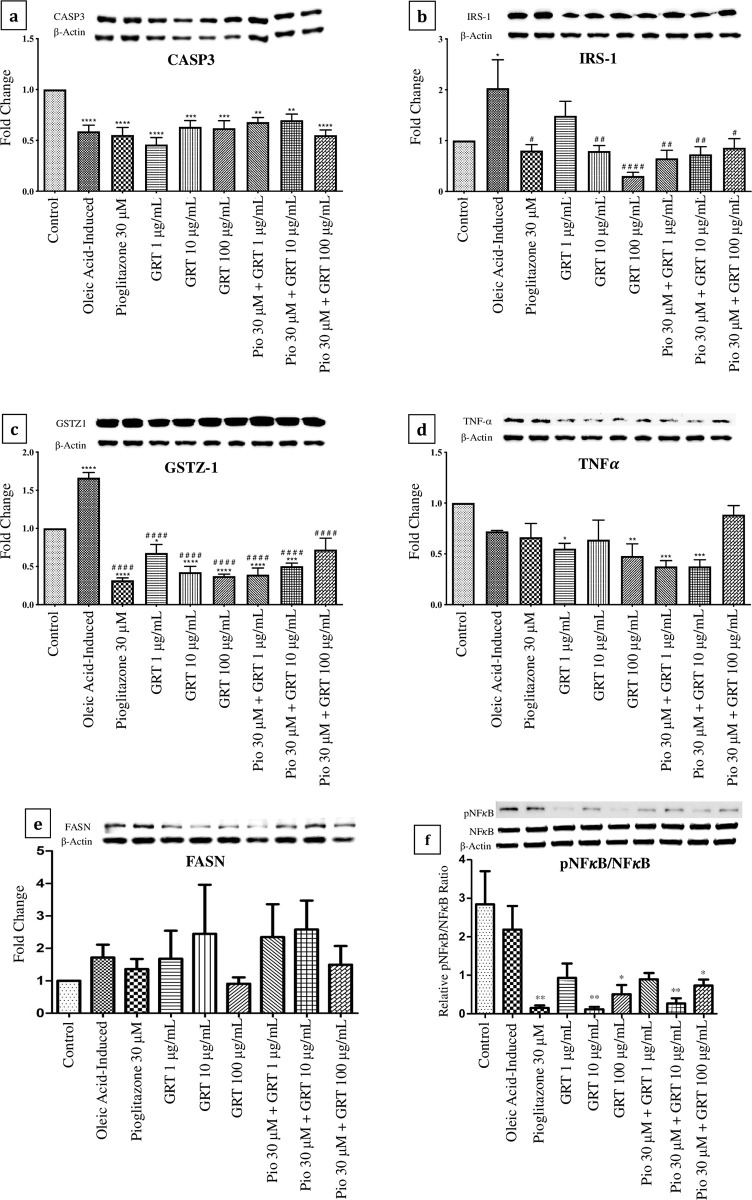
Protein expression of C3A cell-lysates normalized to β-actin, showing the effects of steatosis induction [1 mM oleic acid] and treatment effects of pioglitazone and Afriplex GRT^TM^. Data represents mean ± SEM (*n* = 4), where *P < 0.05, **P < 0.01, ***P < 0.001, ****P < 0.0001 when compared to the control, and ^#^P < 0.05, ^##^P < 0.01, ^####^P < 0.0001 when compared to the oleic acid-induced cells. Histograms represent the protein expressions of; (**a**) Caspase-3, (**b**) IRS-1, (**c**) GSTZ1, (**d**) TNF-α, (**e**) FASN & (**f**) NFκB respectively. **IRS-1—**insulin receptor substrate 1, **GSTZ-1—**glutathione S-transferase zeta 1, **TNF-α -** tumour necrosis factor-alpha, **FASN—**fatty acid synthase, **NFκB**—nuclear factor kappa B.

Caspase-3 protein expression (**Fig 5**) was significantly down-regulated by treatment with oleic acid when compared to the control (p < 0.0001). Subsequent treatment with pioglitazone (30 μM) or Afriplex GRT^TM^ (1 μg/mL, 10 μg/mL & 100 μg/mL) as mono/co-treatments also showed a significant down-regulation in Caspase-3 protein expression when compared to the control (**Fig 5A**). A significant up-regulation in IRS-1 protein expression was observed by treatment with oleic acid when compared to the control (p < 0.05) (**Fig 5B**). Subsequently, a significant down-regulation in IRS-1 protein expression was seen by the various treatments when compared to the oleic acid-induced cells, apart from Afriplex GRT^TM^ (1 μg/mL). A significant up-regulation in GSTZ-1 protein expression was observed by treatment with oleic acid when compared to the control (p < 0.0001) (**Fig 5C**). In contrast, a significant down-regulation in GSTZ-1 protein expression was seen by the various treatments when compared to both normal and oleic acid-induced cells, apart from pioglitazone (30 μM) in combination with Afriplex GRT^TM^ (100 μg/mL), which was only significantly down-regulated when compared to the oleic acid-induced cells. Treatment with Afriplex GRT^TM^ (1 μg/mL) (p < 0.05) and Afriplex GRT^TM^ (10 μg/mL) (p < 0.01) showed a significant down-regulation in TNF-α protein expression when compared to the control (**Fig 5D**). In addition, the combined treatments of pioglitazone 30 μM with Afriplex GRT^TM^ (1 μg/mL) and pioglitazone (30 μM) with Afriplex GRT^TM^ (10 μg/mL), showed a significant down-regulation in TNF-α protein expression when compared to the control (p < 0.001). The full blot images are shown in Figs 1–6 in S1 File Full Western blot images.

## Discussion

Hepatic steatosis results from an increased infiltration of FFAs into the hepatocytes [2]. This increased flux of lipids into the liver cells is also known as fatty liver disease, and in the absence of excessive alcohol use, this disease is termed NAFLD [31]. In order to establish an *in vitro* experimental model of NAFLD, we made use of an optimized concentration of oleic acid to induce steatosis in C3A liver cells. The Oil red O assay in part confirms our *in vitro* NAFLD model with oleic acid-induced cells displaying significantly increased intracellular lipid accumulation when compared to the control. An MTT assay was performed in parallel with the ORO assay, which provided an approximation of the cell viability after exposure of cells to oleic acid. The percentage viability of the cells throughout induction was slightly decreased when compared to the control however, this reduction in MTT activity caused by FFAs has been previously reported in MCF7 cells by Pacheco *et al*. (2018) [32]. Although there was a significant decrease in MTT activity, the treated cells expressed over 70% mitochondrial activity, suggesting that the induction period with oleic acid in the current study was acute when compared to its combination with palmitic acid, as previously seen in a study by Jeong *et al*. (2016) [33].

Relevant to a clinical setting, patients with dyslipidemia and diabetes experience increased levels of FFAs and glucose in circulation, amongst other risk factors involved in the development and pathogenesis of NAFLD [34]. In fact, SREBF1 and ChREBP are major transcriptional regulators that induce key lipogenic enzymes, such as FASN, to promote lipogenesis in the liver [35]. In addition to ChREBP regulating the expression of genes related to glucose and lipid metabolism, literature has shown that this transcriptional factor helps induce hepatic steatosis, in combination with other related complications like dyslipidemia and glucose intolerance [35, 36]. In the current study, the mRNA expression of transcriptional regulators *SREBF1* and *ChREBP*, were both down-regulated by treatment with pioglitazone and Afriplex GRT™, corresponding to the reduction in lipid content seen with the ORO data. Interestingly, the combined use of pioglitazone and Afriplex GRT™ treatment showed an even greater reduction in lipid content suggesting that there may have been an additive effect by combining the treatment regiments. This confirms some of our previous findings [37], we have reported, that bioactive compounds from rooibos like aspalathin can improve the therapeutic properties of antidiabetic drugs such as a metformin in improving glucose control and reducing fat content in diabetic mice, when compared to using monotherapies. Nonetheless, evidence from the current study indicates that the mechanism by which Afriplex GRT™, especially when combined with pioglitazone, reduces hepatic lipid content could possibly be explained by the suppression of transcriptional regulators that induce lipogenic enzymes [37].

Conventionally, lipid accumulation in hepatocytes is a result of obesity as well as insulin resistance causing an increased release of FFAs from adipocytes [38]. The rise in insulin and glucose levels in the circulation triggers *de novo* lipogenesis, contributing to the abnormal retention of FFAs in hepatocytes [39, 40]. Briefly, when insulin binds to the insulin receptor substrate its tyrosine kinase is activated through phosphorylation, subsequently activating the protein kinase B (AKT) pathway [41, 42]. Insulin also stimulates the AKT pathway by the up-regulation of FASN thereby increasing hepatic lipogenesis [42, 43]. Oleic acid up-regulated IRS-1 at both the mRNA and protein level thereby stimulating the up-regulation of *FASN* downstream, confirming the increase of hepatic lipid content with the oleic acid-induced cells. Subsequent treatment with pioglitazone and Afriplex GRT™ down-regulated the mRNA and protein expression of IRS-1. We therefore suggest that the suppressed expression of IRS-1 had caused downstream inhibition of lipogenic enzymes, ultimately reducing hepatic steatosis in our *in vitro* model.

Hepatic oxidative stress is considered a “second hit” in the pathogenesis of fatty liver disease. Main generators of excessive reactive oxygen species in NAFLD include: mitochondrial dysfunction, peroxisomal and microsomal fatty acid oxidation as well as lipid peroxidation [44]. Superoxide dismutase and glutathione peroxidase are antioxidant enzymes that protect against oxidative damage caused by free radical attack [45, 46]. *In vitro* mRNA expression of *SOD2* was significantly down-regulated by treatment with Afriplex GRT™ when compared to the oleic acid-induced cells. At the protein level, GSTZ-1, which is involved in glutathione conjugation reactions, was significantly up-regulated by exposure to oleic acid, and alternatively down-regulated by treatment with pioglitazone and Afriplex GRT™. Although the results showed interventions not to specifically promote *SOD2* mRNA expression levels within this particular experimental model, our results indicate that Afriplex GRT™, either as a monotherapy or in combination with pioglitazone could reduce glutathione conjugation as depicted by decreased protein levels of GSTZ-1. Although analysis of other relevant antioxidant enzymes is necessary, these results signify a strong potential of the extract to suppress oxidative stress to reduce hepatic damage. This hypothesis is supported by our results showing that Afriplex GRT™, especially when used at a higher dose of 100 μg/mL, could reverse detrimental effects linked with cellular damage by improving mitochondrial membrane potential, as measured using JC-1 stain, and reducing caspase-3 release after exposing liver cells to oleic acid. Overall, the current findings infer that treatment with oleic acid induced hepatic oxidative stress *in vitro*, as seen by García-Ruiz *et al*. (2015) [47], and subsequent treatment with Afriplex^TM^ GRT reversed the effects caused by oleic acid.

Beyond the consequences of oxidative stress-induced cellular damage, the cascade of events highlighted by the progression of the disease is linked to the dysregulation of nuclear factor-kappa B, tumor necrosis factor-alpha and interleukin 6, which are key pro-inflammatory cytokines that are essential for functional immune responses [48, 49]. Our study illustrated a down-regulation in TNF-α expression by treatment with Afriplex GRT™ at the protein level. This was expected, as Afriplex GRT™ has been previously reported to have anti-inflammatory properties [24]. We therefore confirm that in this study, in addition to reducing lipid accumulation and ameliorative oxidative stress in liver cells, Afriplex GRT™ may protect against hepatic inflammation by its action on TNF-α or its linked mechanisms [21, 46].

## Conclusion

This study hypothesized that Afriplex GRT™ could mitigate hepatic steatosis and aimed at investigating the hepatoprotective effects of Afriplex GRT™ against hepatic oxidative stress, inflammation, and apoptosis in an *in vitro* model of NAFLD. Oleic acid induced hepatic steatosis in C3A liver cells with a dose-dependent reduction by Afriplex GRT™, more so by its combination with pioglitazone. The mechanism by which Afriplex GRT™ reduced hepatic lipid content in both models was observed by the suppression of transcriptional regulators, *SREBF1* and *ChREBP*, which in turn suppressed the expression of lipogenic enzymes. Improved insulin signaling through the regulation of IRS-1 was also identified as a mechanism of action whereby Afriplex GRT^TM^ ameliorated hepatic steatosis. The current study showed the modulatory benefits of Afriplex GRT^TM^ *in vitro*, highlighting its potential as a therapeutic agent for hepatic homeostasis disturbance in the form of NAFLD. It is difficult to conclude on which Afriplex GRT^TM^ component targets the hepatic homeostasis disturbance, as the extract has a combination of compounds. However, it would be beneficial to explore its therapeutic potential *in vivo*, to provide a more comprehensive perspective.

## Supporting information

S1 File Full Western blot images. (DOCX)

S2 File Gene expression TaqMan probes and qRT-PCR conditions. (DOCX)

S3 File List of measurements, list of abbreviations and table of chemicals/kits. (DOCX)

S4 File List of antibodies. (DOCX)

S5 File Afriplex^TM^ GRT product safety data sheet with physical and chemical properties. (DOCX)
