# Supplementary material for: Afriplex GRTTM extract attenuates hepatic steatosis in an in vitro model of NAFLD
Source: PLoS One. 2024 Apr 17;19(4):e0297572. doi: 10.1371/journal.pone.0297572 (PMC11023570; doi:10.1371/journal.pone.0297572)
Supplement: S1 File — (DOCX) [file pone.0297572.s001.docx]

**S1 Full Western blot images**


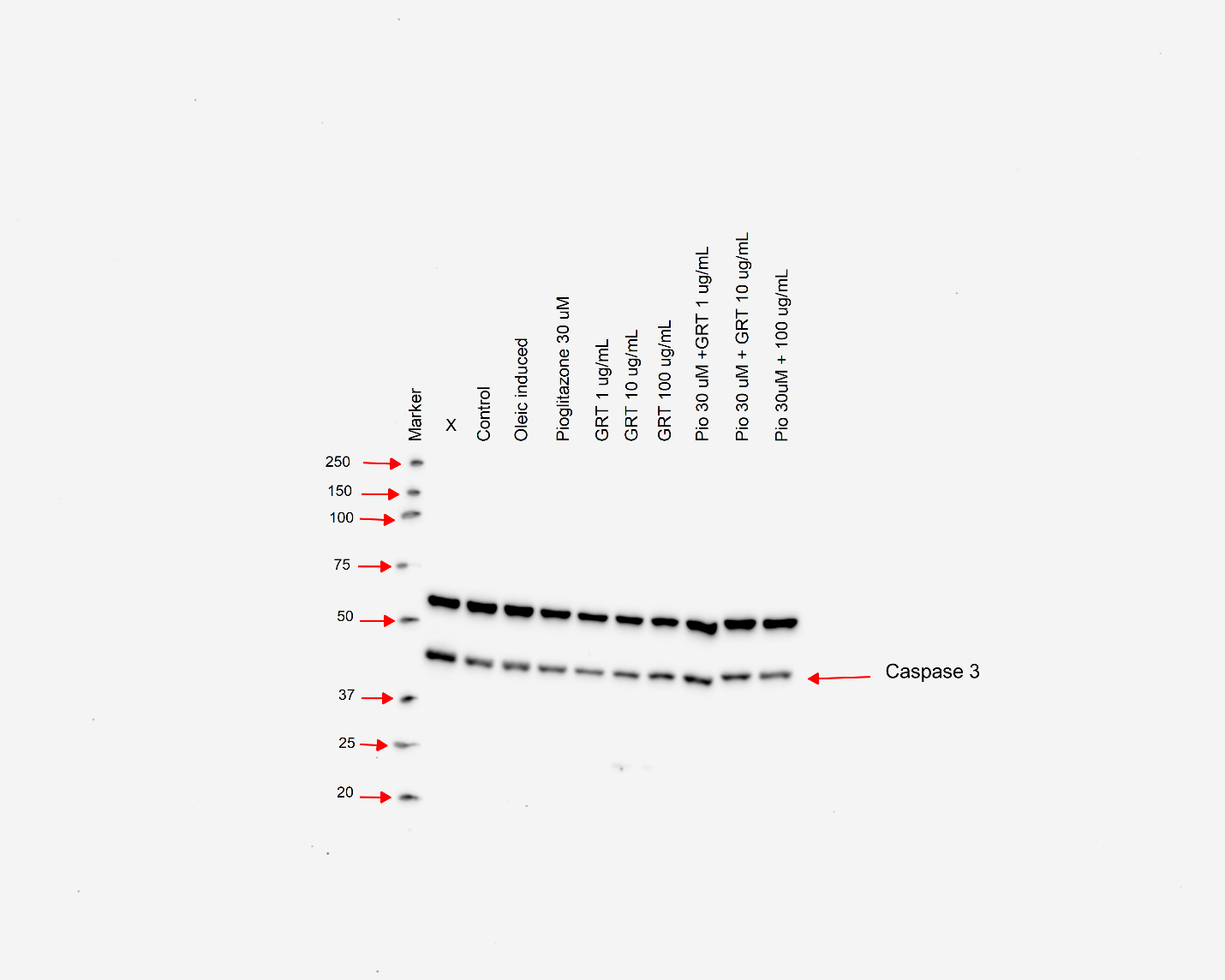


**S1 Fig 1. Caspase 3.** Full blot image of Caspase 3 with the lane marked X (DMSO) not part of the reported data

**
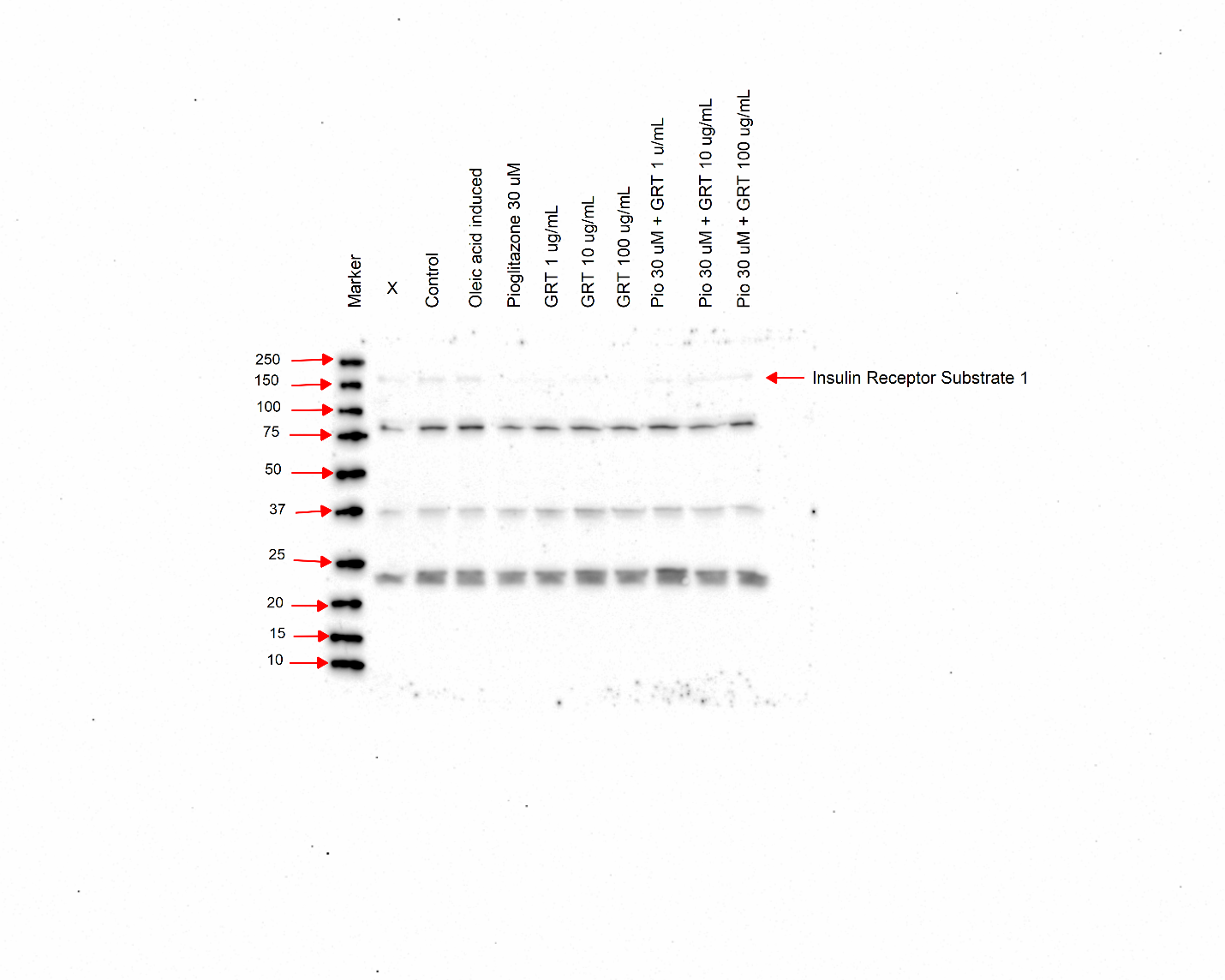
**

**S1 Fig 2. Insulin receptor substrate 1 (IRS-1).** Full blot image of IRS-1 with the lane marked X (DMSO) not part of the reported data


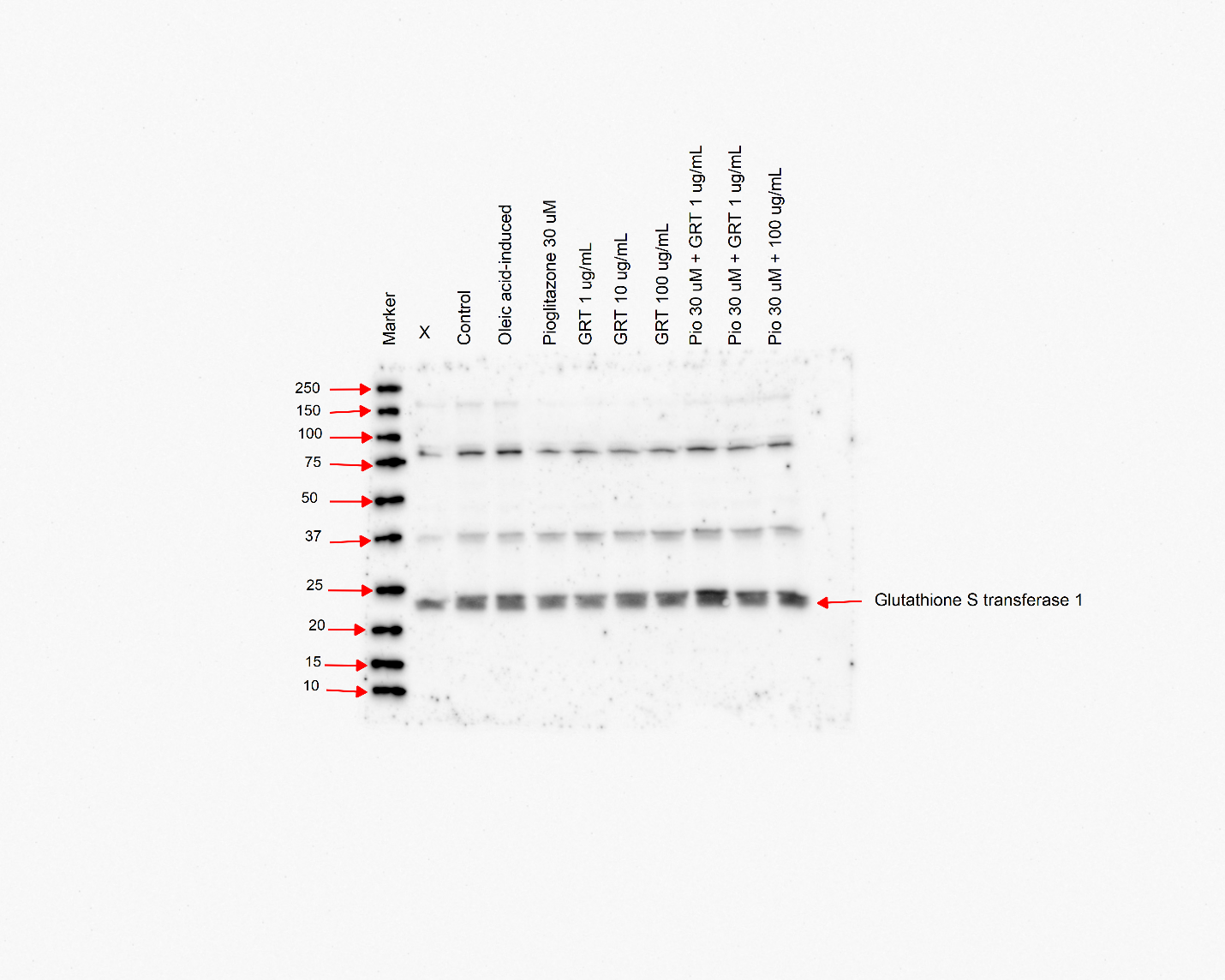


**S1 Fig 3. Glutathione S transferase-1 (GSTZ1).** Full blot of GSTZ1 with the lane marked X (DMSO) not part of the reported data


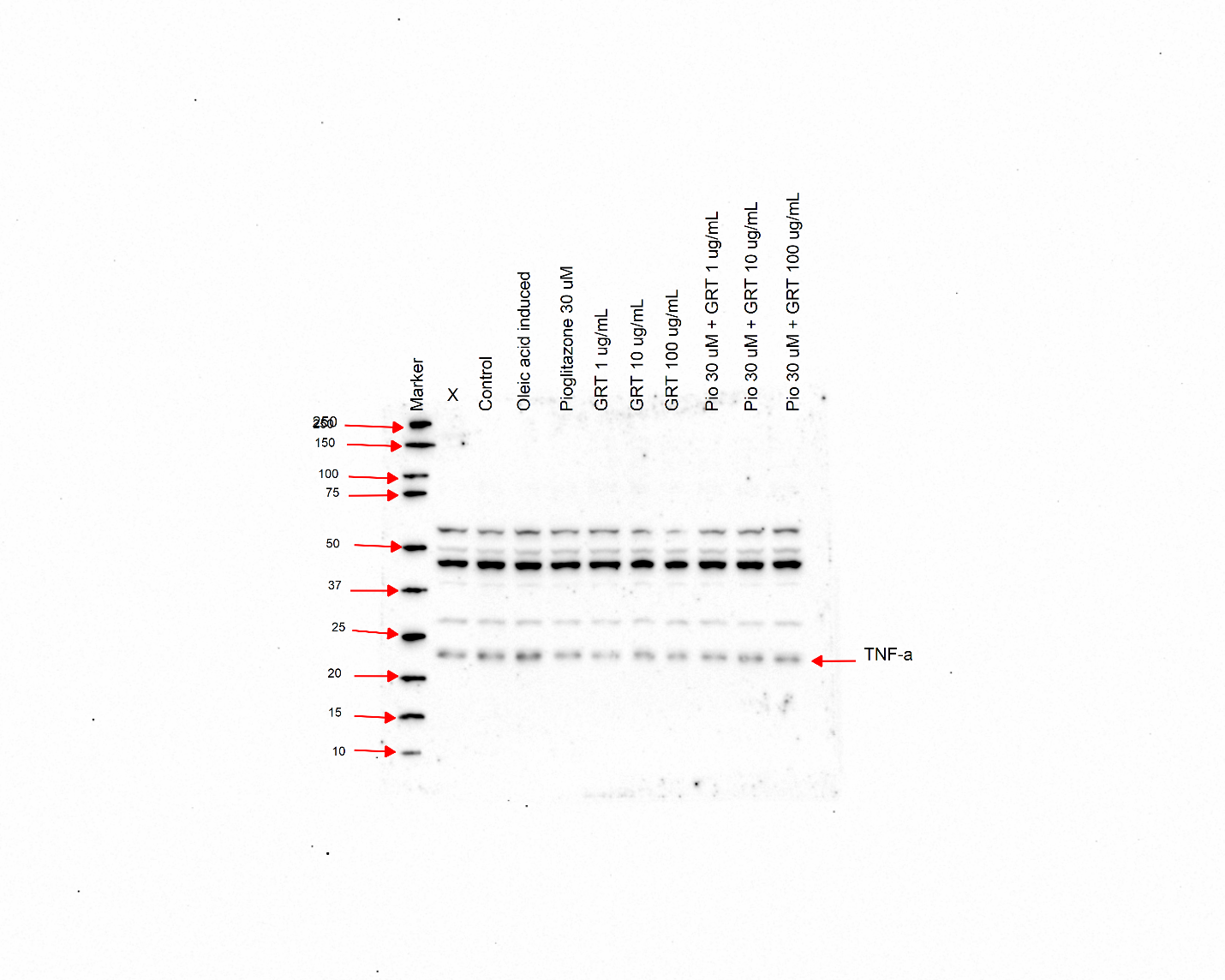


**S1 Fig 4. Tumour necrosis factor alpha (TNF-a).** Full blot image of TNF-a with the lane marked X (DMSO) not part of the reported data


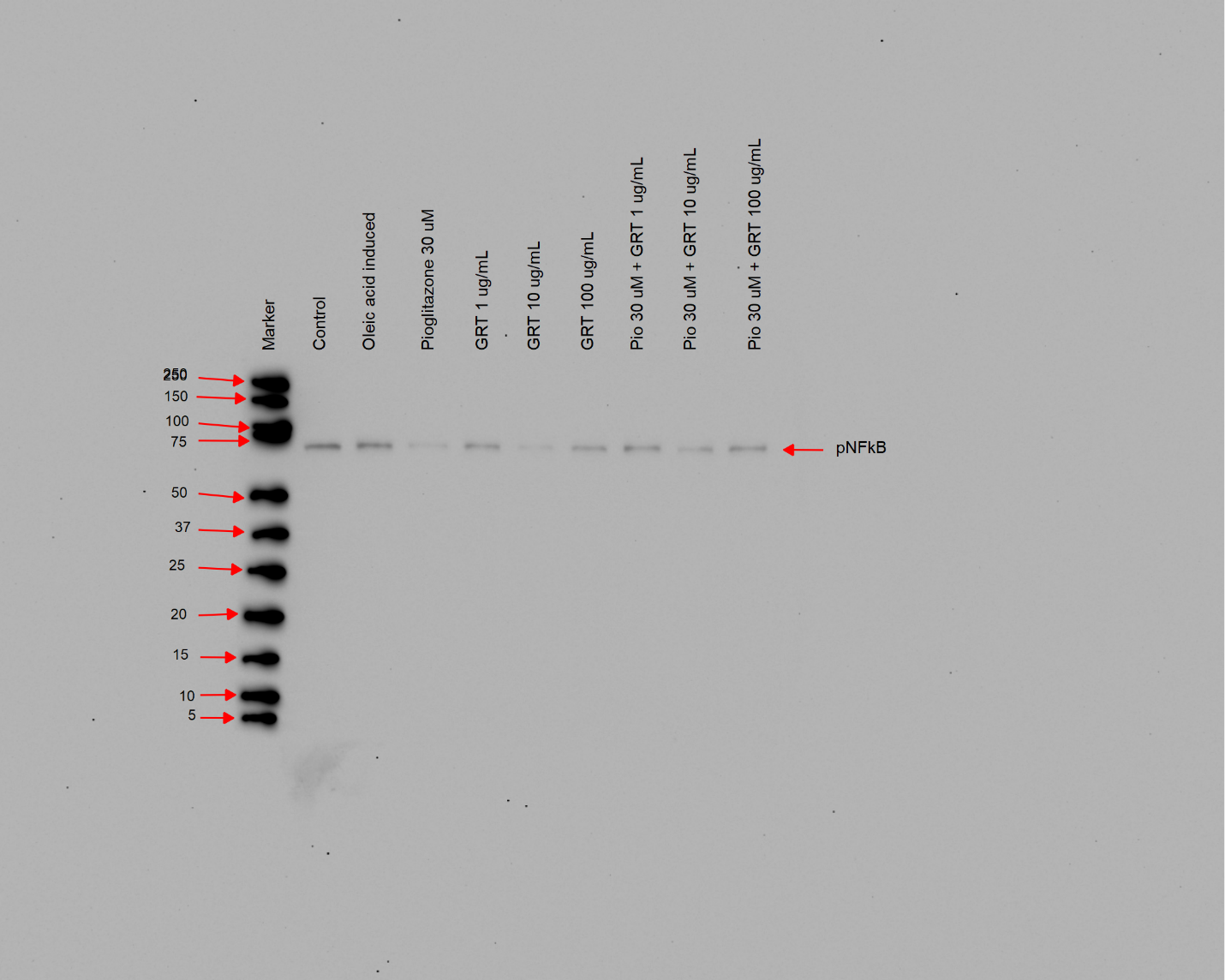


**S1 Fig 5. phosphor-Nuclear factor kappa B (pNFkB).** Full blot image of pNFkB


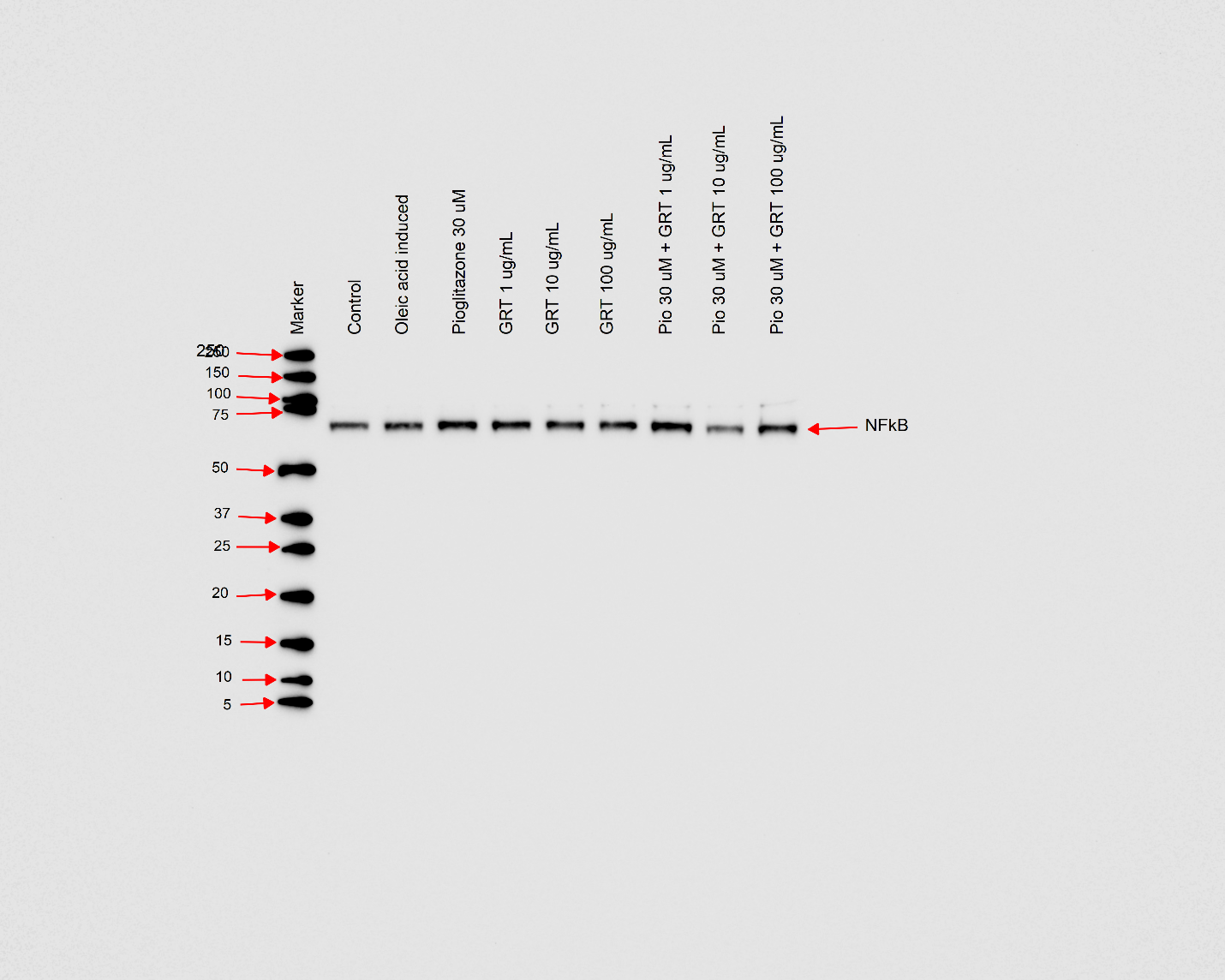


**S1 Fig 6. Nuclear factor kappa B (NFkB).** Full blot image of NFkB
