# Supplementary material for: Afriplex GRTTM extract attenuates hepatic steatosis in an in vitro model of NAFLD
Source: PLoS One. 2024 Apr 17;19(4):e0297572. doi: 10.1371/journal.pone.0297572 (PMC11023570; doi:10.1371/journal.pone.0297572)
Supplement: S2 File — (DOCX) [file pone.0297572.s002.docx]

S2: Gene expression TaqMan probes and qRT-PCR conditions

**S2 Table 1: List of *in vitro* TaqMan® gene expression assay probes**

| **TaqMan® gene expression symbol:** | **TaqMan® gene expression name:** | **Target species:** | **Gene name:** |
| --- | --- | --- | --- |
| MLXIPL | Hs00975714_m1 | Human | *ChREBP* |
| FASN | Hs01005622_m1 | Human | *FASN* |
| IRS1 | Hs00178563_m1 | Human | *IRS-1* |
| SOD2 | Hs00167309_m1 | Human | *SOD2* |
| SREBF1 | Hs02561944_s1 | Human | *SREBF1* |
| ACTB | Hs03023943_g1 | Human | *β-ACTIN* (Housekeeping) |

**S2 Table 2: Thermal Cycler Running Conditions**

| **Stage** | **Repetitions** | **Temperature** | **Time** |
| --- | --- | --- | --- |
| 1 | 1 | 50.0 °C | 02:00 minutes |
| 2 | 1 | 95.0 °C | 10:00 minutes |
| 3 | 40 | 95.0 °C | 00:15 minutes |
|  |  | 60.0 °C | 01:00 minutes |
