# Supplementary material for: Afriplex GRTTM extract attenuates hepatic steatosis in an in vitro model of NAFLD
Source: PLoS One. 2024 Apr 17;19(4):e0297572. doi: 10.1371/journal.pone.0297572 (PMC11023570; doi:10.1371/journal.pone.0297572)
Supplement: S3 File — (DOCX) [file pone.0297572.s003.docx]

# **S3** **List of measurement units, list of abbreviations and table of chemicals/kits**

# **S3 List 1. List of Units of Measurements**

°C Degrees Celsius

hr Hour

mL Millilitres

nm Nanometre

µg Microgram

µL Microliter

µm Micrometre

µM Micromolar

% Percentage

mM Millimolar

mm millimetre

mL Millilitre

mg Milligram

mg/mL Milligram per millilitre

µg/mL Microgram per millilitre

# **S3 List 2. List of Abbreviations**

ANOVA Analysis of variance

ATCC America Type Culture Collection

ATP Adenosine triphosphate

BCA Bicinchoninic acid

BCL-2 B-cell lymphoma 2

BSA Bovine serum albumin

CASP3 Caspase-3

cDNA Complementary deoxyribonucleic acid

ChREBP Carbohydrate-response element-binding protein

CO_2_  Carbon dioxide

CV Crystal violet

DMSO Dimethyl sulfoxide

DNA Deoxyribonucleic acid

EMEM Eagle’s essential minimal medium

FASN Fatty acid synthase

FBS Foetal bovine serum

FFA Free fatty acids

GRT Green rooibos tea

GSTZ1 Glutathione S-transferase Zeta 1

IRS1 Insulin receptor substrate 1

JC-1 5,5',6,6'-Tetrachloro-1,1',3,3' tetraethylbenzimidazolylcarbocyanine iodide

mRNA Messenger ribonucleic acid

MTT 3-[4,5-dimethylthiazol-2-yl]-2,5-diphenyl tetrazolium bromide

NAFLD Non-alcoholic fatty liver disease

NF-κB Nuclear factor-kappa beta

ORO Oil Red O

PAGE Polyacrylamide gel electrophoresis

PBS Phosphate-buffered saline

PCR Polymerase chain reaction

PVDF Polyvinylidene fluoride

qRT-PCR Quantitative real-time polymerase chain reaction

RIPA Radioimmunoprecipitation assay

RNA Ribonucleic acid

SDS Sodium dodecyl sulphate

SOD2 Superoxidase dismutase 2

SREBF1 Sterol regulatory element-binding factor-1

T2DM Type 2 diabetes mellitus

TBST Tris-Buffered Saline and Tween 20

TNF-α Tumour necrosis factor alpha

TZD Thiazolidinedione

VLDL Very low-density lipoprotein

α Alpha

β Beta

γ Gamma

# **S3 Table 1. List of Chemicals/kits**

| **Product (Chemical/Kit)** | **Supplier/Company** |
| --- | --- |
| (ca 12%) Aspalathin-rich Afriplex GRT™ | Afriplex, Paarl, Western Cape, RSA |
| 10x Tris/Glycine/SDS | Bio-Rad, Berkeley, California, USA |
| 12% Mini-PROTEAN® TGX™ Stain-free FastCast Acrylamide kit | Bio-Rad, Berkeley, California, USA |
| 5x TransBlot® Turbo Transfer Buffer, RTA Transfer Kit, LF PVDF | Bio-Rad, Berkeley, California, USA |
| Bovine serum albumin (BSA) – fatty-acid free | Capricorn scientific Auf der Lette 13A, Ebsdorfergrund, Germany |
| C3A human liver cells | American Type Culture Collection, Manassas, USA |
| Carbon dioxide (CO2) | Air Products, Centurion, Gauteng, RSA |
| Clarity™ Western C ECL Substrate | Bio-Rad, Berkeley, California, USA |
| Crystal violet | Merck, Whitehouse Station, NJ, USA |
| Dimethyl Sulfoxide (DMSO) | Sigma-Aldrich, Saint Louis, USA |
| Dulbecco’s phosphate saline buffer (DPBS) | Lonza, Walkersville, MD, USA |
| Eagle’s Minimum Essential Medium (EMEM) | Lonza, Walkersville, MD, USA |
| Ethylenediaminetetraacetic acid disodium salt dihydrate (EDTA) | Sigma-Aldrich, Saint Louis, USA |
| Foetal bovine serum (FBS) | Lonza, Walkersville, MD, USA |
| Invitrogen™ MTT (3-(4,5-Dimethylthiazol-2-yl)-2,5-Diphenyltetrazolium Bromide) | Thermo Fisher Scientific™, Johannesburg, Gauteng, RSA |
| Isopropanol | Sigma-Aldrich, Saint Louis, USA |
| L-Glutamine | Lonza, Walkersville, MD, USA |
| Low fat free milk powder | Clover, Johannesburg, Gauteng, RSA |
| LumiGLO Chemiluminescent Substrate Kit | Cell Signaling Technology, Boston, USA |
| Methanol | VWR Chemicals, Fontenay-sous-Bois, France |
| Oil O Red | Sigma-Aldrich, Saint Louis, USA |
| Pierce™ BCA Protein Assay Kit | Thermo Fisher Scientific™, Johannesburg, Gauteng, RSA |
| Ponceau S Stain | Sigma-Aldrich, Saint Louis, USA |
| Precision Protein™ StrepTactin-HRP Conjugate | Bio-Rad, Berkeley, California, USA |
| Protease Inhibitors | Roche, Basel, Switzerland |
| Restore Plus Western blot stripping buffer | Thermo Fisher Scientific™, Johannesburg, Gauteng, RSA |
| Sodium dodecyl sulphate (SDS) | Sigma-Aldrich, Saint Louis, USA |
| Sodium hydroxide (NaOH) | Sigma-Aldrich, Saint Louis, USA |
| Trypan blue | Invitrogen, Carlsbad, CA, USA |
| Trypsin-versene | Lonza, Walkersville, MD, USA |
| Tween-20 | Sigma-Aldrich, Saint Louis, USA |
| β-mercaptoethanol | Sigma-Aldrich, Saint Louis, USA |
