# Supplementary material for: Afriplex GRTTM extract attenuates hepatic steatosis in an in vitro model of NAFLD
Source: PLoS One. 2024 Apr 17;19(4):e0297572. doi: 10.1371/journal.pone.0297572 (PMC11023570; doi:10.1371/journal.pone.0297572)
Supplement: S4 File — (DOCX) [file pone.0297572.s004.docx]

# **S4 List of Antibodies**

# **S4 Table 1. List of Antibodies**

| **Protein name** | **Reference/**  **Supplier** | **Source** | **Cat#** | **Pathway** | **Mwt** |
| --- | --- | --- | --- | --- | --- |
| Anti-Mouse | Cell Signalling Technology, Danvers, MA, USA | Goat | 14709 | Secondary antibody | - |
| Anti-Rabbit | Cell Signalling Technology, Danvers, MA, USA | Goat | 14708 | Secondary antibody | - |
| β-Actin | Santa Cruz Biotechnology, Dallas, Texas, USA | Mouse | sc-47778 | Loading Control Protein | 43 kDa |
| [casp3] Caspase 3 | Cell Signalling Technology, Danvers, MA, USA | Rabbit | 9962 | Apoptosis | 35 kDa |
| [fasn] Fatty Acid Synthase | Cell Signalling Technology, Danvers, MA, USA | Rabbit | 3180 | Fatty Acid Synthesis | 272 kDa |
| [gstz1] Glutathione Transferase Zeta 1 | Abcam, Cambridge, UK | Rabbit | ab153995 | Oxidative Stress | 24 kDa |
| [irs-1] Insulin Receptor Substrate 1 | Cell Signalling Technology, Danvers, MA, USA | Rabbit | 3407 | Insulin signalling | 131 kDa |
| [nfκb] Nuclear Factor-kappa B | Cell Signalling Technology, Danvers, MA, USA | Rabbit | 8242 | Inflammatory Response | 65 kDa |
| [pnfκb] phospho-Nuclear Factor-kappa B | Cell Signalling Technology, Danvers, MA, USA | Rabbit | 3033 | Inflammatory Response | 65 kDa |
| [tnfα] Tumour Necrosis Factor alpha | Cell Signalling Technology, Danvers, MA, USA | Rabbit | 11948 | Inflammatory Response | 17 kDa |
