# Supplementary material for: Afriplex GRTTM extract attenuates hepatic steatosis in an in vitro model of NAFLD
Source: PLoS One. 2024 Apr 17;19(4):e0297572. doi: 10.1371/journal.pone.0297572 (PMC11023570; doi:10.1371/journal.pone.0297572)

# **S5** **Afriplex GRT^TM^ product safety data sheet with physical and chemical properties**

Full copy at: <https://www.afriplexgrt.com/images/pdfs/>[SDS-CPE03287-03 Afriplex GRT extract-final.pdf](https://www.afriplexgrt.com/images/pdfs/SDS-CPE03287-03%20Afriplex%20GRT%20extract-final.pdf)


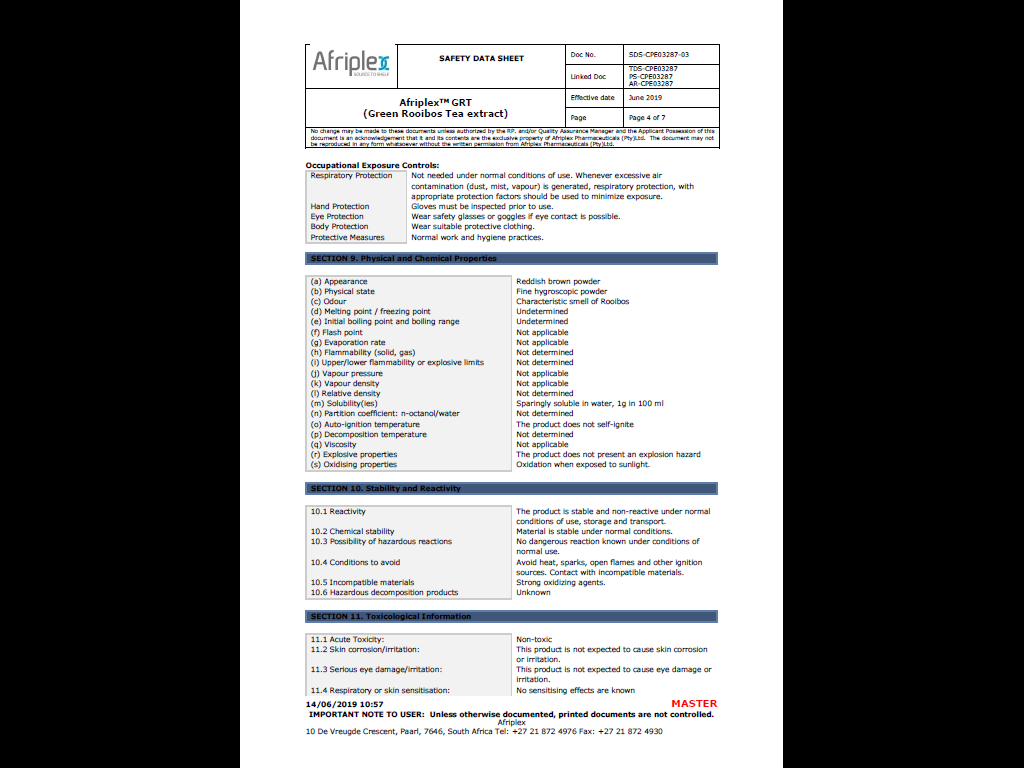

Supplement: S5 File — (DOCX) [file pone.0297572.s005.docx]
